# A “Forbidden Fruit Effect”: An Eye-Tracking Study on Children’s Visual Attention to Food Marketing

**DOI:** 10.3390/ijerph17061859

**Published:** 2020-03-13

**Authors:** Alice Binder, Brigitte Naderer, Jörg Matthes

**Affiliations:** 1 Advertising and Media Effects Research Group, Department of Communication, University of Vienna, Währinger Straße 29, 1090 Vienna, Austria; joerg.matthes@univie.ac.at; 2 Department of Media and Communication, Ludwig-Maximilians-University Munich; Oettingenstraße 67, 80538 Munich, Germany; Brigitte.Naderer@ifkw.lmu.de

**Keywords:** healthy and unhealthy food marketing, public health, children, eye-tracking, pupil dilation, parents, candy prohibition

## Abstract

Obesity in children is an international health concern. Against this background, there is an increasing interest in understanding how healthy and unhealthy food marketing in narrative media can affect children. In particular, children’s implicit reactions, such as visual attention and emotional arousal, are far from being sufficiently understood. We conducted an eye-tracking study, presenting children one of two versions of a narrative media-stimulus, either presenting an unhealthy food (i.e., candy condition; *N* = 34), or a healthy food (i.e., fruit condition; *N* = 34). As dependent variables, we investigated dwell time (i.e., visual attention) and pupil dilation (i.e., emotional arousal). As moderators, we included children’s prohibition of candy at home and children’s level of BMI in our models. Our results indicate that mean dwell time did not differ between conditions and that the moderators did not exert any effect. Moreover, pupil dilation did not differ between conditions but was moderated by parents’ candy prohibition at home (η_p_^2^ = 0.080). The results show that children who are not allowed to consume candy at home react with higher emotional arousal when exposed to candy placements than children allowed to eat candy at home. Thus, depending on children’s contextual factors, children react differently to unhealthy food cues.

## 1. Introduction

Obesity and overweight in children are international health concerns. The number of overweight and obese children worldwide has reached an alarming 41 million [1]. In this regard, media use has been connected to childhood obesity [2]. First of all, media use is a passive way of spending one’s time, and therefore, it replaces active free-time activities [3]. Thus, overall screen time is associated with sedentary behaviors and overall low levels of fitness [4]. Therefore, the behavior media consumption per se is deeply related to factors that are fostering obesity because it increases sedentary behavior and decreases physical activity. Additionally, food marketing depicts a high number of products high in fat, salt, and sugar [5]. Moreover, a lot of persuasive marketing techniques for especially unhealthy products are used [6,7,8], which has been found to serve as a trigger for children’s unhealthy food choices [9,10,11,12,13,14,15]. To counter the effects of unhealthy food marketing in the media, there is an increasing interest in understanding how healthy food depictions can affect children’s food choices. The empirical evidence available to date suggests that healthy food marketing is less powerful in shaping children’s consumption behavior compared to unhealthy ones [16,17,18,19]. However, the mechanisms behind these differences are far from being sufficiently understood.

Previous studies on food placements as a marketing technique have mainly focused on explicit measurements, such as interviews regarding children’s food preferences [20]. These measurements are vulnerable to social desirability that often reduces predictive validity [21,22]. Implicit reactions are not regulated on purpose [23] and may reveal important insights that cannot be obtained with conventional methods [24]. Eye-tracking has the potential to shed light on audiences’ spontaneous reactions and perception processes [25]. This method, therefore, allows to track participants eye movements and allows for a rather unconscious measure of the participants’ attention and reactivity concepts. In addition, it is rather non-inversive, as participants are able to receive media content as they typically would, e.g., while viewing a movie or pictures on a computer screen [23]. In this regard, many measurements are possible. Visual attention is measured by dwell time, which indicates how long the gaze lasts on a product. An increase in dwell time is generally associated with heightened attention for the presented products [26]. Pupil dilation is commonly interpreted as an indicator of emotional arousal. Food cues that activate us should show larger deviations from our non-activated pupil than food cues that do not trigger an emotional response [27,28]. If food cues lead to emotional arousal, this may be an indicator of either a strong like or dislike of the depicted food [23].

In the Reactivity of Embedded Food Cues in Advertising model (REFCAM), Folkvord and colleagues [29] describe that food cues within media-content influence children’s physiological and psychological responses, such as visual reactions [10]. These responses are referred to as cue reactivity. To fully understand cue reactivity toward food cues, many determinates are important. On the message side, the type of food which is integrated within editorial content may influence children’s cue reactivity [30].

Only a few studies we are aware of have tested the effects of healthy versus unhealthy foods on visual attention [23,31,32,33]. First, Graham and colleagues [23] measured visual attention of overweight and normal weight women toward unhealthy sweet, unhealthy savory, and healthy foods. They did not find any group differences in the average amount of time spent gazing at these described products. However, these findings cannot be generalized to children because of their low levels of inhibitory control [34]. Second, Ogle and colleagues [31] compared the visual attention of children for pictures of unhealthy versus healthy foods. Children paid more visual attention to unhealthy foods. Both studies used static pictures and did not take the attentive potential of food cues embedded into a narrative media-content into account. In fact, static pictures cannot be compared to narrative media. In narrative media, the foods are depicted in the context of a narrative plot. In order to understand and enjoy narrative media, children allocate their attention to the story. When using static pictures, by contrast, children most likely allocate their full attention to the depicted foods. Two studies showed some evidence that children’s visual attention might be affected, depending on the nutritional value a food has. Spielvogel and colleagues [33] tested with a within-subject design of how children react to different food cues. This study presented that children show higher visual attention to unhealthy compared to healthy food cues. However, Naderer and colleagues [32] tested with a between-subject design of how children react to healthy, unhealthy, or non-edible products. In this case, the authors did not find any effects on dwell time when comparing the two food conditions. Taken together, the available evidence does not allow clear conclusions about how children allocate visual attention to healthy and unhealthy foods embedded in narrative media.

The literature on self-regulation suggests that exposure to short-term temptations, for instance, unhealthy food cues, activates an eating enjoyment goal, which in turn triggers attentional bias for unhealthy food cues [35]. It is assumed that children are commonly less able to ignore short-term temptations [36] based on the immaturity of their attentional flexibility. We assume that children are less able to shift their attention away from affect-based cues, such as unhealthy food cues embedded within media-content [37]. We hypothesize that children will allocate more visual attention to unhealthy food cues compared to healthy food cues (H1).

Studies investigating pupil diameter changes have been mainly conducted in the 1960s and 1970s and have investigated the effects on adults only and not in the area of health topics [38]. Thus, recent research in this area is scarce. There is no known study that uses this measurement when investigating children’s reactions. However, effect studies clearly suggest that children show larger behavioral responses when exposed to unhealthy compared to healthy food cues [14,15]. Therefore, it seems likely that children react with higher emotional arousal toward unhealthy compared to healthy products (H2).

There is hardly any knowledge about the factors that determine the relationship between healthy and unhealthy food depictions and children’s visual attention and pupil dilation. Based on extant literature, we propose two important moderators. First, parents can use different strategies to control their children’s eating behavior; for example, restrictions of specific foods [39]. Restrictions are employed quite frequently by parents, especially regarding unhealthy foods [30]. However, studies show that the simple restriction of foods may lead to an emotional response, namely, children’s reactance against these rules [40]. This may be explained through the so-called “forbidden fruit effect”. It describes that anything which seems to be unavailable is, as a result, more desirable. The effect of breaking the rules imposed on us by others is associated with the reactance and commodity theory [41]. The reactance theory assumes that people like to behave according to their own desires, and if this freedom is threatened, they experience reactance, that is, negative emotional states that humans want to avoid [42]. To escape the emotional unpleasantness of reactance, people behave against the rules forced upon them [43]. If parents forbid a specific food group at home, it may be possible that children desire this specific food even more, which leads to more visual attention or emotional arousal for the forbidden food (RQ1).

Second, some studies have demonstrated that children’s BMI influences how food is perceived [44,45]. Thus, children may show more visual attention and pupil dilation in response to unhealthy foods with rising levels of BMI [21]. Since, to date, no studies on BMI status and visual reactions of children exist, we want to investigate this research gap with this study (RQ2).

**Figure 1:**
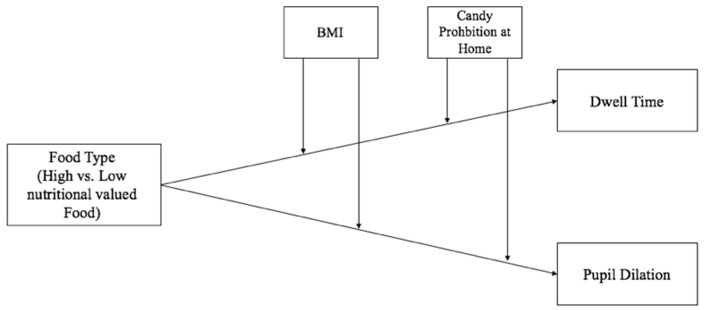
Conceptual model.

For the hypothesized model, see Figure 1.

Overall, this study contributes to this important research field in investigating children’s dwell time and pupil dilation in reaction to an unhealthy versus a healthy product. Studies exploring the effects on children did not measure pupil dilation [31,32,33]. However, this important measurement could shed light on children’s emotional reactions toward different food cues [28]. Moreover, as stated in the REFCAM [28], individual susceptibility factors such as food restrictions at home [30] or the BMI [46] are crucial. However, studies with children did not take these factors into account [31,32,33].

## 2. Materials and Method

### 2.1. Design

We conducted an experimental between-subject study with two conditions, combining survey data with eye-tracking measurements. We collected the data in a primary school in Austria. The Regional Education Authority of the participating school approved the study. For each child, we furthermore obtained the parents’ written consent and the children’s oral consent. We exposed children to one of two different versions of a self-created, narrative media-stimulus in which either a mandarin (i.e., fruit condition) or a fruit gum (i.e., candy condition) was integrated. In a pre-test with another sample of children, we made sure that children see a fruit gum as an unhealthy snack (*N* = 40; unhealthy: 87.5 %) and a mandarin as a healthy snack (*N* = 40; healthy: 97.5 %).

Initially, a total of 75 children between 6 and 11 years took part in the study. We had to exclude two children because of problems in the stimulus presentation and another five children who showed poor deviation results following final calibration (>1°). A complete dataset of *N* = 68 children (Mage = 8.18; *SD* = 1.46; 50.0% female) remained. The sample size is in line with other eye-tracking studies in this area of research [12,21].

The children completed individual eye-tracking sessions. We randomly assigned each participant to the fruit (*n* = 34) or candy condition (*n* = 34), with one experimenter supervising the eye-tracking sessions. The children did not know that their eye movements would be tracked. They only knew that they were watching a short audio-visual cartoon and that, afterwards, they would be asked to answer a few short questions. After the stimulus presentation, each child was led to a separate interview room and was interviewed individually by an independent experimenter. Afterwards, we measured the children’s height and weight. Then we brought children back into their class. At the end of the experiment, all children were extensively debriefed about the purpose of the study as well as the advantages of eating healthy.

### 2.2. Stimuli

We designed two versions of an audio-visual media-stimulus by creating a narrative cartoon-story (6:00 min) using the software POWTOON (Powtoon Ltd, London, San Francisco, UK, US). Our stimulus presented the story of two twin pandas who found a treasure map. So as not to go hungry on their treasure hunt, they bring their favorite snack (mandarins = fruit condition; fruit gums = candy condition) along (see Appendix A). We saved the stimuli at a resolution of 972 × 1137 pixels. Images had onscreen dimensions of approximately 40 cm in height and 34 cm in width and appeared on a 17-inch monitor. In both conditions, the marketing placement was integrated five times for 20,800 ms. In four instances out of five, the character mentioned the specific product name in the accompanying sound-track (“I need a break, and a mandarin/a fruit gum”). We kept the stimulus identical in both conditions, only varying the target product [16].

### 2.3. Measures

As the dependent variables, we measured participants’ dwell time and pupil diameter changes with an eye-tracking system (SMI iView X™ RED, SensoMotoric Instruments GmbH, Teltow, Germany) with high spatial resolution and a sampling rate of 120 Hz. Although viewing was binocular, only the right eye movements were monitored [47]. At the beginning of the experiment, we conducted a 5-point calibration test and a 5-point validation test at the end of each session.

Moreover, to calculate dwell time and pupil diameter changes, first of all, the areas of interests (AOIs) were defined. Mandarins and fruit gums within the media-stimulus were defined as the AOIs [32]. We calculated the mean dwell time for all AOIs for each picture, and we computed the dwell time means for each condition [25]. Values ranged from 835.94 to 6191.07 ms (*M* = 3576.10; *SD* = 1164.82) for the mean dwell time of the embedded food cues.

For measures of pupil diameter changes, change scores were calculated [24]. In the current study, pupil diameter changes were calculated relative to the pupil diameter during the validation task. The validation task shows only a white surface with a red focus point. We thus used this neutral picture as the baseline for the rest of the arousing and colorful story. Moreover, for the validation task, the eye-tracker measured pupil diameter many times, creating a good baseline for children’s individual average of the not-aroused state of the pupil. Pupil diameter changes for the AOIs ranged from −0.66 to 3.56 mm (*M* = 1.04; *SD* = 0.78).

To estimate whether children’s parents allow their children to eat candy at home, we asked children after the stimulus presentation an open-ended question: ‘Are you allowed to eat candy at home?’. We later dummy-coded these answers (“0 = No”, “1 = Yes”; 66.2%, *n* = 45). When children answered indirectly with a “Yes” or “No”, we asked a second time if they would tend more to “Yes” or “No” regarding general situations at home. A recent meta-analysis on a similar topic showed that children are overall able to report about their diet very well because children’s perceptions correlated with independent, validated reports [48]. We are mainly interested in children’s subjective impression of eating rules at home because perceived norms such as social approval about a specific behavior from, for example, parents are drivers for a specific behavior [49].

For children’s BMI, we measured children’s size and weight and the standard deviation score of BMI (zBMI) was computed to adjust for age and sex (*M* = 0.51; *SD* = 1.10; [50]). The results show that 38.2% (*n* = 26) of all children in this study had a BMI score above the cut-offs of normal weight and are characterized as overweight (+1SD; 29.4%) or obese (+2SD; 8.8%) [1].

### 2.4. Randomization and Manipulation Checks

A randomization check for gender (χ² = 0.00, df = 1, Φ = 0.00, n.s.), age (F(1, 66) = 0.03, n.s.), candy prohibition at home (F(1, 66) = 0.58, n.s.), and zBMI (F(1, 66) = 0.24, n.s.) was successful. Moreover, zBMI, pupil dilation, and dwell time were normally distributed.

Both food options were similar in size and color to make sure that, if differences in the measurements occur, they can only be led back to the fact that one product is healthy (mandarin) and one is unhealthy (fruit gum; see Appendix A). To additionally assure that children were able to recognize which snack was presented within the media-stimulus, the character mentioned the specific product name four times out of five visual presentations. To assess whether children noticed the difference between the two products, we measured whether children correctly remembered what product was shown within the stimulus. For the candy condition, 91.2% (*n* = 31) correctly identified the fruit gum; likewise, 91.2% (*n* = 31) of all children in the fruit condition identified the mandarin. The manipulation check was successful (*p* < 0.001).

Moreover, to make sure that for all children, regardless of their age, the stimuli are similarly appealing, we asked children after the stimulus presentation how they would evaluate the cartoon which they just watched (1= really bad; 4 = really good; *M* = 3.59, *SD* = 0.63). There was no correlation between children’s age and evaluation of the stimuli (*r* = −0.197, *p* = 0.108).

### 2.5. Data Analysis

First of all, we calculated the mean dwell time for the embedded foods: In the candy condition, the mean dwell time on the integrated fruit gum was 3526.45 ms (*SD* = 1135.94); in the fruit condition the mean dwell time was 3626.75 ms (*SD* = 1208.01). In the next step, we looked at the main effects of the condition on dwell time and pupil dilation. We then calculated a one-way ANOVA for dwell time as the dependent variable, and a repeated-measures general linear model for pupil diameter changes as the dependent variable. Then, we included candy prohibition at home and children’s levels of BMI as moderators for both dependent variables. Based on the norms in social science, we define a significant effect if the probability of error is under 5% (*p* < 0.05).

## 3. Results

First, we looked at the main effects of the experimental conditions on children’s dwell time and pupil diameter change. In the candy condition, the mean dwell time on the presented fruit gum was 3526.45 ms (*SD* = 1135.94); in the fruit condition, the mean dwell time was 3626.75 ms (*SD* = 1208.01). No main effect of the conditions on dwell time was found (H1; F(1, 67) = 0.12, *p* = 0.728, η2 = 0.002).

For pupil diameter, we calculated a repeated measures general linear model to demonstrate if children’s pupil diameter changed on the basis of the validation tasks, comparing the AOIs of the two conditions. No main effects of the conditions on pupil diameter changes were present (H2; F(1, 66) = 1.57, *p* = 0.890, η2 = 0.000).

We included BMI status as a possible moderator. BMI did not moderate children’s mean dwell time (RQ1; F(3, 64) = 0.13, *p* = 0.730, η2 = 0.002) or pupil diameter change (RQ1; F(3, 64) = 108.52, *p* = 0.534, η2 = 0.006) when comparing our two conditions.

The moderator candy prohibition at home did not influence children’s mean dwell time comparing the two conditions (RQ2; F(3, 64) = 0.19, *p* = 0.358, η2 = 0.013). There was, however, a significant effect of pupil diameter changes in the two conditions when taking candy prohibition at home into account (RQ2; F(1, 64) = 5.53, *p* = 0.022, η2 = 0.080; for all results, see Table 1).

**Figure 2:**
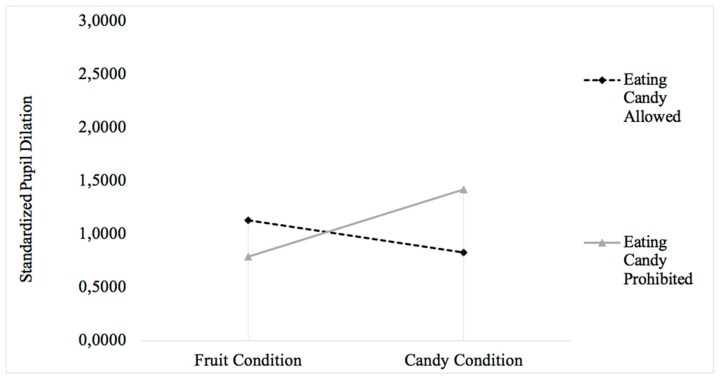
Interaction effect of the experimental conditions and candy prohibition regarding children’s standardized pupil dilation.

To investigate if the moderator had the same influence in both conditions, we conducted a t-test for the pupil diameter changes. The results show that candy prohibition at home only showed a significant influence in the candy condition (eating candy prohibited: *M* = 1.42, *SD* = 0.72; eating candy allowed: *M* = 0.83, *SD* = 0.54; t(32) = 2.71, *p* = 0.011). Whether sweets were forbidden at home did not impact the children’s pupil dilation in the fruit condition (eating candy prohibited: *M* = 0.79, *SD* = 0.84; eating candy allowed: *M* = 1.12, *SD* = 0.91; t(32) = −1.01, *p* = 0.320). When children’s parents forbid children to eat candy at home and children were exposed to candy cues within a media context, the children’s pupil dilation was significantly increased compared to children in the candy condition who are allowed to eat candy at home. For a visualization of the effect, see Figure 2.

## 4. Discussion

This study aims to investigate children’s implicit reactions toward healthy and unhealthy food cues within a narrative media context using eye-tracking measures. For the first time in this area of research, we measured children’s pupil dilation. Moreover, we included important individual susceptibility factors, such as BMI and candy prohibition at home.

Our study cannot confirm that unhealthy foods lead to higher visual attention or pupil dilation compared to healthy foods. These results discount studies using static pictures of unhealthy and healthy foods [31]. When using static pictures, the full attention is channeled to the specific picture. In contrast, when using narrative media, the foods are embedded within the stimulus, which may lead to less attention toward the food cues. The more natural narrative setting shows no differences between unhealthy and healthy foods concerning children’s visual attention and pupil dilation. This is in line with a study using a similar design [32]. As an alternative explanation, one could argue that the candy and the fruit were not clearly distinguishable. However, nearly all children were able to correctly classify the candies and fruits as unhealthy and healthy.

In contrast to studies that demonstrated the moderating role of the BMI on adults’ emotional arousal [20] and children’s orofacial reactions [45], children’s BMI levels did not influence the mean dwell time or the changes in pupil diameter in this study. Since only one product was presented within the narrative stimulus, it is possible that children’s BMI would play a role if children had to choose what to look at, thus, if healthy and unhealthy products are presented within one stimulus.

We found that parental restrictions of unhealthy foods at home impacted children, regarding their level of emotional arousal. This may indicate a “forbidden fruit effect” [41]. Children’s pupil diameter increased for children in the candy condition when they were not allowed to eat candy at home compared to children who were allowed to eat candy at home. These effects do not occur in the fruit condition. An explanation for the rise in the pupil size might be that the pupil size increases due to negative emotions to a product banned at home and presumably disapproved of by children’s parents. However, since the biological instinct drives humans to unhealthy products [30], and overall unhealthy products are connected with immediate reward [51], this explanation seems unlikely. In this context, the increase in the pupil might be more likely interpreted as a higher liking of the product since it seems unlikely that children show a negative emotional response toward unhealthy products. However, these findings could also be due to reverse causation. Perhaps parents who do not allow their children to eat candy at home have that rule because, for those children, candy is more appealing and they tend to overeat it, whereas parents do not need such a rule if their children do not find candy as tempting. Nevertheless, pupil dilation was not influenced by candy prohibition at home (*p* = 0.525). Another explanation for the increase in pupil diameter for children who are not allowed to eat candy at home is provided by the incentive sensation theory. This theory describes that the presentation of food cues leads to the impression of the availability of this specific food group [52]. It seems plausible that this impression affects children’s emotional arousal, especially when children are normally not allowed to eat these foods. How this emotional arousal then translates into behavioral preference has to be investigated further. Our results indicate that parent’s rules for eating habits at home may have the potential to reveal interesting insights in this research area. In this regard, we only measured the restriction of unhealthy foods at home. Factors such as how parents communicate these food rules [17], parents’ nutritional knowledge [53], or meal preparation at home [54] might also be important contextual factors that should be investigated in further research. Since these first results point to the direction that parental rules are important, further research should investigate this in more detail.

The mean dwell time was not influenced by this specific eating rule at home. This can be explained by the fact that both stimuli are highly similar and easy to process. The candy and the mandarin have the same size, almost the same color, and the same shape, and both stimuli are far from being complex. It can also be assumed that children are familiar with both products. Following this reasoning, there is no reason to look longer on the candy even though children are not allowed to eat it.

As implications for food marketing research, we would argue that overall unhealthy and healthy foods lead to similar unconscious reactions. However, depending on children’s contextual factors, this reaction might differ. Since the forbidden unhealthy product led to more emotional arousal, we can conclude that placing food cues as something that is forbidden might be a good marketing strategy because children might perceive these products as more desirable. However, this strategy can only be effective for unhealthy foods. Therefore, it is important that marketers are aware of the possible negative effects of unhealthy foods placed within audio-visual media. Overall, marketers should place more healthy foods within children’s media to contribute to public health.

### Limitations and Further Research

Our research faces some limitations. First, since this was the first study to include pupil dilation measures with children, it is important to replicate these results. Second, future studies should take the whole proposed process of the REFCAM [28] into account. Investigating the whole model was not possible in our study due to the limited sample size. However, for further research, it is important to demonstrate how different food groups within media-stimuli influence children’s visual attention and pupil reactions, and how these reactions, in turn, influence children’s actual food choices. Thus, connecting implicit reactions with real behavior might shed light on how processing specific messages influences real eating behavior. Since the behavior itself causes health problems, this seems highly important. Some studies point toward the effect that the mere presence of food leads to a choice of the unhealthier option [15,16]. The implicit reactions might be similar for both food types, which is supported by our results [32]. Third, we only compared one unhealthy product and one healthy product. Therefore, we can only make conclusions for these specific snacks since food preference may play a major role. Further research can demonstrate if other products lead to the same effects.

## 5. Conclusions

All in all, in narrative media, the presentations of unhealthy foods do not automatically lead to higher visual attention or emotional arousal. Yet parent’s restrictions of candy at home influences children’s emotional arousal toward unhealthy products. Rephrased, these restrictions can backfire and lead to higher emotional arousal when confronted with unhealthy products. Based on an increase in emotional arousal, audio-visual media may prompt children to consume unhealthy snacks when there are restrictions at home. On a methodological level, we conclude that implicit measurements do reveal important insights into how health messages can influence audience reactions. It is, therefore, extremely important to conduct more eye-tracking experiments with children to fully understand which processes are taking place during media consumption with integrated food cues. Such studies may also shed light on how children’s attention can be triggered for healthy foods.

## Figures and Tables

**Table 1:** Main and interaction effects explaining dwell time and pupil dilation for food cues.

Main & Interaction Effects	Dwell Time				Pupil Dilation			
	df	F	η_p_^2^	*p*	df	F	η_p_^2^	*p*
Food Type	1.00	0.12	0.002	0.728	1.00	1.57	0.000	0.890
Food Type × BMI	3.00	0.13	0.002	0.730	3.00	108.52	0.006	0.534
Food Type × Candy Prohibition at Home	3.00	0.19	0.013	0.358	1.00	5.53	0.080	0.022

## Appendix A

**Candy Condition**

**Fruit Condition**

**Presented Food Cues within in the Candy Condition (left) and in the Fruit Condition (right):**

## References

[1] (2016). World Health Organization—WHO. Obesity and Overweight. Factsheet June 2016. http://www.who.int/mediacentre/factsheets/fs311/en/.

[2] Fuller-Tyszkiewicz M., Skouteris H., Hardy L.L., Halse C. (2012). The associations between TV viewing, food intake, and BMI. A prospective analysis of data from the Longitudinal Study of Australian Children. Appetite.

[3] Carson V., Hunter S., Kuzik N., Gray C.E., Poitras V.J., Chaput J.-P., Saunders T.J., Katzmarzyk P.T., Okely A.D., Connor Gorber S. (2016). Systematic review of sedentary behaviour and health indicators in school-aged children and youth: An update. Appl. Physiol. Nutr. Metab..

[4] Chinapaw M.J.M., Proper K.I., Brug J., van Mechelen W., Singh A.S. (2011). Relationship between young peoples’ sedentary behaviour and biomedical health indicators: A systematic review of prospective studies: Childhood sitting and health. Obes. Rev..

[5] Matthes J., Naderer B. (2019). Sugary, fatty, and prominent: Food and beverage appearances in children’s movies from 1991 to 2015. Pediatric Obes..

[6] Halford J.C.G., Boyland E.J. (2013). The marketing of foods and non-alcoholic beverages to children. Setting the research agenda. Appetite.

[7] Radnitz C., Byrne S., Goldman R., Sparks M., Gantshar M., Tung K. (2009). Food cues in children’s television programs. Appetite.

[8] Kemps E., Tiggemann M., Hollitt S. (2016). Longevity of attentional bias modification effects for food cues in overweight and obese individuals. Psychol. Health.

[9] Brown K.A., Ogden J., Vögele C., Gibson E.L. (2008). The role of parental control practices in explaining children’s diet and BMI. Appetite.

[10] Folkvord F., Anschütz D.J., Wiers R.W., Buijzen M. (2015). The role of attentional bias in the effect of food advertising on actual food intake among children. Appetite.

[11] Harris J.L., Bargh J.A., Brownell K.D. (2009). Priming effects of television food advertising on eating behavior. Health Psychol..

[12] Harris J.L., Speers S.E., Schwartz M.B., Brownell K.D. (2012). US food company branded advergames on the Internet: Children’s exposure and effects on snack consumption. J. Child. Media.

[13] Matthes J., Naderer B. (2015). Children’s consumption behavior in response to food product placements in movies: Children’s consumption behavior and food product placements. J. Consum. Behav..

[14] Matthes J., Naderer B. (2016). Product placement disclosures: Exploring the moderating effect of placement frequency on brand responses via persuasion knowledge. Int. J. Advert..

[15] Naderer B., Matthes J., Zeller P. (2018). Placing snacks in children’s movies: Cognitive, evaluative, and conative effects of product placements with character product interaction. Int. J. Advert..

[16] Folkvord F., Anschütz D.J., Buijzen M., Valkenburg P.M. (2013). The effect of playing advergames that promote energy-dense snacks or fruit on actual food intake among children. Am. J. Clin. Nutr..

[17] Naderer B., Matthes J., Binder A., Marquart F., Mayrhofer M., Obereder A., Spielvogel I. (2018). Shaping children’s healthy eating habits with food placements? Food placements of high and low nutritional value in cartoons, Children’s BMI, food-related parental mediation strategies, and food choice. Appetite.

[18] Naderer B., Binder A., Matthes J., Mayrhofer M. (2020). Healthy, sweet, brightly colored, and full of vitamins: cognitive and affective persuasive cues of food placements and children’s healthy eating behavior. Int. J. Advert.

[19] Binder A., Naderer B., Matthes J. (2019). Do children’s food choices go with the crowd? Effects of majority and minority peer cues shown within an audiovisual cartoon on children’s healthy food choice. Soc. Sci. Med..

[20] Czyzewska M., Graham R., Ceballos N.A., Preedy R.V., Watson R.R., Martin C.R. (2011). Explicit and implicit attitudes to food. Handbook of Behavior, Food and Nutrition.

[21] Kemps E., Tiggemann M. (2015). Approach bias for food cues in obese individuals. Psychol. Health.

[22] Czyzewska M., Graham R. (2008). Implicit and explicit attitudes to high- and low-calorie food in females with different BMI status. Eat. Behav..

[23] Graham R., Hoover A., Ceballos N.A., Komogortsev O. (2011). Body mass index moderates gaze orienting biases and pupil diameter to high and low calorie food images. Appetite.

[24] Scheiter K., van Gog T. (2009). Using eye tracking in applied research to study and stimulate the processing of information from multi-representational sources. Appl. Cognit. Psychol..

[25] King A.J., Bol N., Cummins R.G., John K.K. (2019). Improving visual behavior research in communication science: An overview, review, and reporting recommendations for using eye-tracking methods. Commun. Methods Res..

[26] Robinson T. (1993). The neural basis of drug craving: An incentive-sensitization theory of addiction. Brain Res. Rev..

[27] Andreassi J.L. (2013). Psychophysiology: Human Behavior & Physiological Response.

[28] Bradley M.M., Miccoli L., Escrig M.A., Lang P.J. (2008). The pupil as a measure of emotional arousal and autonomic activation. Psychophysiology.

[29] Folkvord F., Anschütz D.J., Boyland E., Kelly B., Buijzen M. (2016). Food advertising and eating behavior in children. Curr. Opin. Behav. Sci..

[30] Birch L.L., Fisher J.A. (1995). Appetite and eating behavior in children. Pediatric Clin. North Am..

[31] Ogle A.D., Graham D.J., Lucas-Thompson R.G., Roberto C.A. (2017). Influence of cartoon media characters on children’s attention to and preference for food and beverage products. J. Acad. Nutr. Diet..

[32] Naderer B., Binder A., Matthes J., Spielvogel I., Forrai M. (2020). Food as an eye-catcher. An eye-tracking study on Children’s attention to healthy and unhealthy food presentations as well as non-edible objects in audiovisual media. Pediatric Obes..

[33] Spielvogel I., Matthes J., Naderer B., Karsay K. (2018). A treat for the eyes. An eye-tracking study on children’s attention to unhealthy and healthy food cues in media content. Appetite.

[34] John D.R. (1999). Consumer socialization of children: A retrospective look at twenty-five years of research. J. Consum. Res..

[35] Papies E.K., Stroebe W., Aarts H. (2008). The allure of forbidden food: On the role of attention in self-regulation. J. Exp. Soc. Psychol..

[36] Kerr A., Zelazo P.D. (2004). Development of “hot” executive function: The children’s gambling task. Brain Cogn..

[37] Rozendaal E., Lapierre M.A., van Reijmersdal E.A., Buijzen M. (2011). Reconsidering advertising literacy as a defense against advertising effects. Media Psychol..

[38] Hess E.H., Greenfield N.S., Sternbach R.A. (1972). Pupillometrics: A method of studying mental, emotional and sensory processes. Handbook of Psychophysiology.

[39] Birch L.L., Fisher J.O., Grimm-Thomas K., Markey C.N., Sawyer R., Johnson S.L. (2001). Confirmatory factor analysis of the Child Feeding Questionnaire: A measure of parental attitudes, beliefs and practices about child feeding and obesity proneness. Appetite.

[40] Jansen E., Mulkens S., Jansen A. (2007). Do not eat the red food!: Prohibition of snacks leads to their relatively higher consumption in children. Appetite.

[41] Gosselt J.F., De Jong M.D.T., Van Hoof J.J. (2012). Effects of Media Ratings on Children and Adolescents: A Litmus Test of the Forbidden Fruit Effect. J. Commun..

[42] Brehm J.W., Strull T.K. (1989). Psychological reactance: Theory and applications. NA-Advances in Consumer Research.

[43] Engs R., Hanson D.J. (1989). Reactance Theory: A Test with collegiate drinking. Psychol. Rep..

[44] Jansen A., Theunissen N., Slechten K., Nederkoorn C., Boon B., Mulkens S., Roefs A. (2003). Overweight children overeat after exposure to food cues. Eat. Behav..

[45] Soussignan R., Schaal B., Boulanger V., Gaillet M., Jiang T. (2012). Orofacial reactivity to the sight and smell of food stimuli. Evidence for anticipatory liking related to food reward cues in overweight children. Appetite.

[46] Dixon H., Scully M., Niven P., Kelly B., Chapman K., Donovan R., Martin J., Baur L.A., Crawford D., Wakefield M. (2014). Effects of nutrient content claims, sports celebrity endorsements and premium offers on pre-adolescent children’s food preferences: Experimental research: Child responses to food pack promotions. Pediatric Obes..

[47] Henderson J.M., Weeks P.A., Hollingworth A. (1999). The effects of semantic consistency on eye movements during complex scene viewing. J. Exp. Psychol. Hum. Percept. Perform..

[48] Merson B., Pezdek K., Saywitz K. (2017). A meta-analysis of children’s self-reports of dietary intake. Psychol. Health.

[49] Rivis A., Sheeran P. (2003). Descriptive norms as an additional predictor in the theory of planned behaviour: A meta-analysis. Curr. Psychol..

[50] De Decker A., Sioen I., Verbeken S., Braet C., Michels N., De Henauw S. (2016). Associations of reward sensitivity with food consumption, activity pattern, and BMI in children. Appetite.

[51] Birch L.L. (1999). Development of food preferences. Annu. Rev. Nutr..

[52] Robinson T.E., Berridge K.C. (2008). The incentive sensitization theory of addiction: Some current issues. Philos. Trans. R. Soc. B Biol. Sci..

[53] Gibson E.L., Wardle J., Watts C.J. (1998). Fruit and vegetable consumption, nutritional knowledge and beliefs in mothers and children. Appetite.

[54] Futrell Dunaway L., Carton T., Ma P., Mundorf A., Keel K., Theall K. (2017). Beyond Food Access: The Impact of Parent-, Home-, and Neighborhood-Level Factors on Children’s Diets. IJERPH.

